# Supplementary material for: Motor imagery involves predicting the sensory consequences of the imagined movement
Source: Nat Commun. 2018 Apr 24;9:1617. doi: 10.1038/s41467-018-03989-0 (PMC5915435; doi:10.1038/s41467-018-03989-0)
Supplement: Supplementary file 1 — Supplementary Information [file 41467_2018_3989_MOESM1_ESM.pdf]

Supplementary Information

Motor imagery involves predicting the sensory consequences of the imagined movement

Kilteni *et al.*

Supplementary Note 1.

We performed an additional control experiment (Experiment 3) to test the hypothesis that the imagery-driven attenuation observed in Experiment 1 requires that participants imagine moving their right index finger at the same time that they receive the external stimulation on their left index finger. Introducing a delay between the real movement and its somatosensory consequence in the force-matching task is known to eliminate sensory attenuation[1]. Thus, we expected that the same would hold true for motor imagery. To address this issue, we designed an experiment consisting of four conditions, three of them being the same as the conditions in Experiment 1 (base, press and imagine). In the fourth, novel condition ($imagine_{delay}$), the participants were asked to imagine pressing their right index finger against the sensor on top of their left index finger as they had done in the imagine condition, but the reference force was presented with a temporal delay after the imagery task had finished. Specifically, each trial in the $imagine_{delay}$ condition started with the participants imagining pressing 2 N for 3 seconds. In a short training session just before this condition, the participants practiced actually pressing 2 N with their right index finger so they would have a recent experience of how hard they should imagine pressing. After the 3 seconds of motor imagery, the participants were verbally instructed to stop imagining the movement and remove their right index finger from the sensor. The reference force was then applied for 3 seconds. Finally, the participants were asked to reproduce the reference force with the slider. The interval between the end of the imagery phase and the application of the reference force was 5 seconds in this condition in order for the experimenter to provide verbal instructions and for the participants to stop the imagery task and remove their finger from the sensor.

Twelve healthy naïve participants (5 women and 7 men, 11 right-handed and 1 ambidextrous) aged 18-30 years old were recruited for Experiment 3, none of whom had participated in any of the previous experiments. As in Experiments 1 and 2, each condition in Experiment 3 included 35 trials, and the order of conditions was randomized. In Experiment 3, the participants were blindfolded in all conditions. We did not expect that blindfolding the participants would affect the force-matching task in the base and press conditions, but we decided to match this factor across all conditions in Experiment 3. As in the previous experiments, we recorded electromyography (EMG) from the right first dorsal interosseous (FDI) muscle to ensure that the participants fully relaxed their index finger while imagining. Finally, at the end of each of the two imagery conditions, we administered the same questionnaire as used in Experiments 1 and 2 to assess the self-rated imagery performance (see **Main Text**).

To determine if the participants attenuated the reference forces, we performed planned pairwise comparisons for the forces the participants reproduced in the press, imagine, and $imagine_{delay}$ with those in the base condition. As in the previous experiments, to determine whether participants had their right index finger relaxed in the imagery conditions, we performed planned comparisons for the root-mean-square (RMS) EMG activity of the right FDI averaged during all of the reference forces (base, press, imagine) or during all the periods when the participants were imagining ($imagine_{delay}$). Finally, Wilcoxon signed-rank tests were used to test the differences in the participants' ratings of imagery difficulty and vividness. All comparisons were two-tailed unless stated otherwise.

The results of Experiment 3 are summarized in **Supplementary Fig. 1**. As seen in **Supplementary Fig. 1a-c**, the participants reproduced weaker forces when they previously pressed (mean ± SD = 1.888 ± 0.285 N) or imagined pressing (mean ± SD = 1.867 ± 0.249 N) compared to the forces in the base condition (mean ± SD = 2.065 ± 0.324 N) and the $imagine_{delay}$ condition (mean ± SD = 2.034 ± 0.333 N). As expected, the forces produced with the slider when the participants had previously pressed (press) were significantly weaker than the forces in the base condition ($t(11) = -1.91$, $p = 0.041$, CI = [-Inf, -0.011], one-tailed), replicating the basic attenuation effect during overt movements. Moreover, a planned pairwise comparison between the imagine and base conditions replicated the imagery-induced sensory attenuation effect from Experiment 1 ($t(11) = -2.67$, $p = 0.022$, CI = [-0.362, -0.035]). Critically, however, the forces participants produced when the reference force was applied after the imagery task ($imagine_{delay}$ condition) were of a similar magnitude to those produced during the base condition, with no significant difference between conditions ($t(11) = -0.32$, $p = 0.755$, CI = [-0.245, 0.183]). Moreover, a Bayes factor analysis for this comparison suggested that the observed data were 3.33 times more likely to have occurred under the null hypothesis than under the alternative hypothesis ($BF_{10} = 0.30$ ±0.02%, default Cauchy prior width r = 0.707). These findings suggest that the efference copy generated by the motor imagery needs to occur at the same time as the tactile stimulation in order for the latter to be attenuated, effectively mirroring the temporal constraint of somatosensory attenuation during overt self-touch [1,2].

As expected, the FDI activity was greater during the press condition (mean ± SD = 0.036 ± 0.018 V) than during the base (mean ± SD = 0.002 ± 0.0005 V), the imagine condition (mean ± SD = 0.002 ± 0.0005 V) and the $imagine_{delay}$ condition (mean ± SD = 0.002 ± 0.0005 V) (**Supplementary Figure 1d**). Pairwise comparisons confirmed that there was comparable muscular activity between the base and imagine conditions ($t(11) = 1.17$, $p = 0.266$, CI = [-0.00008, 0.0003]), the base and $imagine_{delay}$ conditions ($t(11) = 0.69$, $p = 0.506$, CI = [-0.0002, 0.0004]), and the imagine and $imagine_{delay}$ conditions ($t(11) = 0.12$, $p = 0.905$, CI = [-0.0003, 0.0003]), while the activity in each of these three conditions differed significantly from that in the press condition (all p-values < 0.001). Finally, a Wilcoxon signed-rank test revealed no significant differences between the participants' ratings of difficulty ($n = 12$, $V = 16$, $p = 0.454$) and vividness ($n = 12$, $V = 11$, $p = 0.419$) of the two imagery conditions (**Supplementary Figure 1e,f**).

Supplementary Figure.

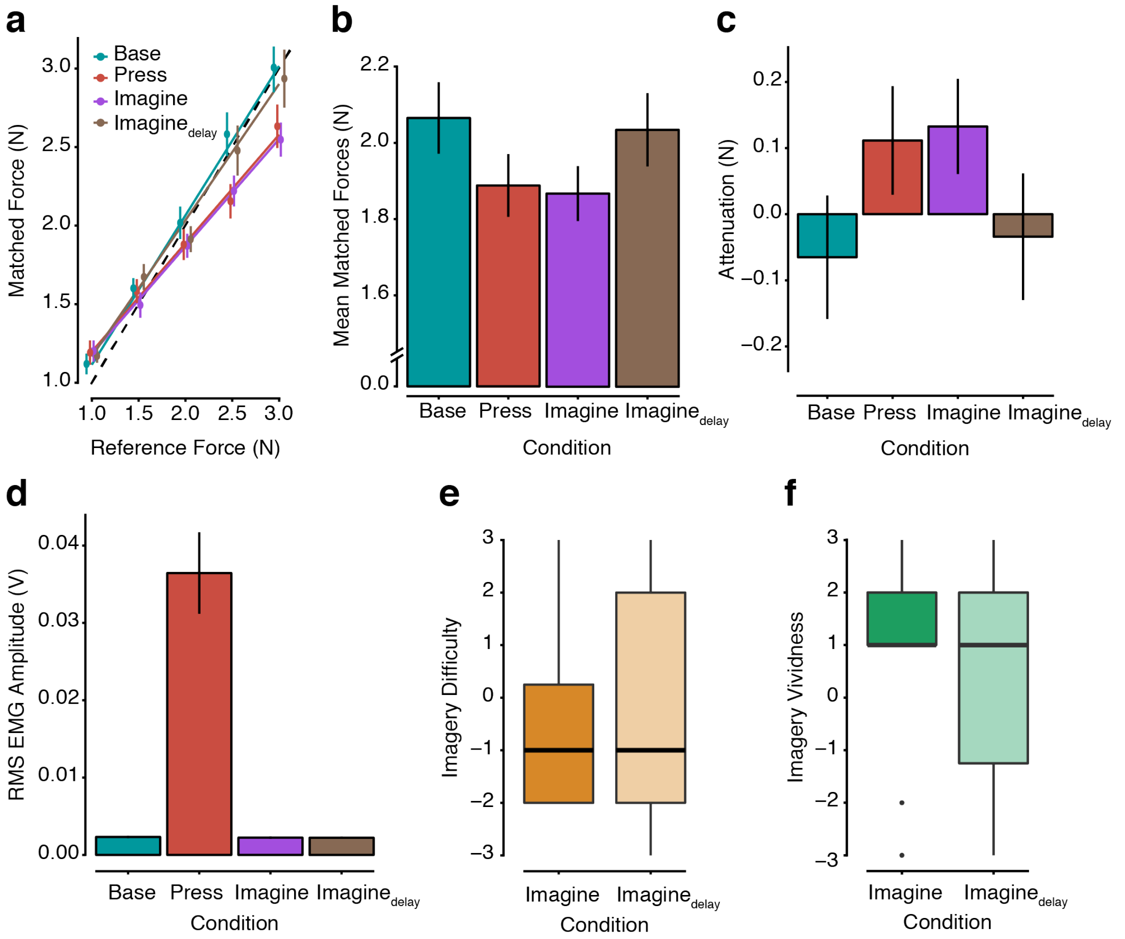

Supplementary Figure 1. Results from Experiment 3. a, Forces generated by participants (matched forces) as a function of the reference force (mean ± SE). **b**, Mean matched forces displayed per condition (mean ± SE). The forces in the imagine condition were significantly weaker than those in the base condition, replicating the imagery-induced attenuation effect of Experiment 1. In contrast, no significant differences were detected between the base and imagine$_{delay}$ conditions, suggesting the absence of attenuation when a delay is inserted between the imagery task and the reference force. **c**, Somatosensory attenuation expressed as the difference between the reference forces and the matched forces per condition (mean ± SE). **d**, Root-mean-square EMG activity of the right FDI muscle per condition (mean ± SE). No significant differences were detected between the base, imagine and imagine$_{delay}$ conditions, showing that the participants were able to relax their right index finger in both motor imagery conditions. **e, f,** Boxplots for the ratings of difficulty and vividness of the imagine and imagine$_{delay}$ conditions. Data beyond the end of the whiskers are plotted as individual data points. No significant differences were detected between the ratings of the two conditions.

Supplementary References.

1. Bays, P. M., Wolpert, D. M. & Flanagan, J. R. Perception of the consequences of self-action is temporally tuned and event driven. *Curr. Biol.* **15,** 1125–1128 (2005).
2. Blakemore, S. J., Frith, C. D. & Wolpert, D. M. Spatio-temporal prediction modulates the perception of self-produced stimuli. *J. Cogn. Neurosci.* **11,** 551–559 (1999).
